# A rapid and simple endoscopic resection of gastric fundus submucosal tumors

**DOI:** 10.1002/deo2.70006

**Published:** 2024-08-27

**Authors:** Mingwen Guo, Bing Yang, Yi Juan Guo, WenGuang Yang, SiChao Wen, YuHong Ren

**Affiliations:** ^1^ Department of Gastroenterology Qionglai Medical Center Hospital Qionglai China

**Keywords:** cost saving, endoscopic submucosal dissection, gastric submucosal tumors, rapid endoscopic resection, simple endoscopic resection

## Abstract

A 56‐year‐old male patient was diagnosed with a submucosal tumor in the fundus of the stomach. The conventional operation method is endoscopic submucosal dissection. We present a case of rapid tumor resection without employing traditional endoscopic submucosal dissection instruments such as a mucotomy knife and endoscopic injection needle, resulting in substantial cost savings for the patients.

## INTRODUCTION

Gastric submucosal tumors (SMTs) are frequently encountered stromal tumors, with a notable prevalence in the gastric fundus. Gastrointestinal stromal tumors (GISTs) are the most common mesenchymal neoplasms, accounting for 1%–2% of all neoplasms of the digestive tract. The differential diagnosis includes the other gastric subepithelial lesions, including lipoma, neuroendocrine tumor, leiomyoma, neural stromal tumors (schwannoma, neuroma, and neurofibroma), ectopic pancreas (pancreatic rest), and extrinsic compression.^1^

Gastric SMTs, often originating from the muscular layer, traditionally posed challenges for endoscopic submucosal dissection (ESD), leading to a preference for surgical resection. The development of laparoscopic and endoscopic cooperative surgery and subsequent endoscopic techniques, including endoscopic full‐thickness resection and endoscopic selective muscular dissection, have expanded treatment options.^2^ The methods mentioned above have drawbacks such as trauma, lengthy procedures, complexity, and high costs.^3^ In particular, due to the special anatomical location of the gastric fundus, accessing the lesions with the endoscopic tip can be challenging, which significantly increases the difficulty of ESD.^4^ Although some Band‐assisted endoscopic mucosal resection techniques have alleviated the difficulty, numerous challenges persist.^5^ In the report, we present a case of rapid gastric fundus SMT resection without employing traditional ESD instruments such as a mucotomy knife and endoscopic injection needle.

## CASE REPORT

**FIGURE 1:**
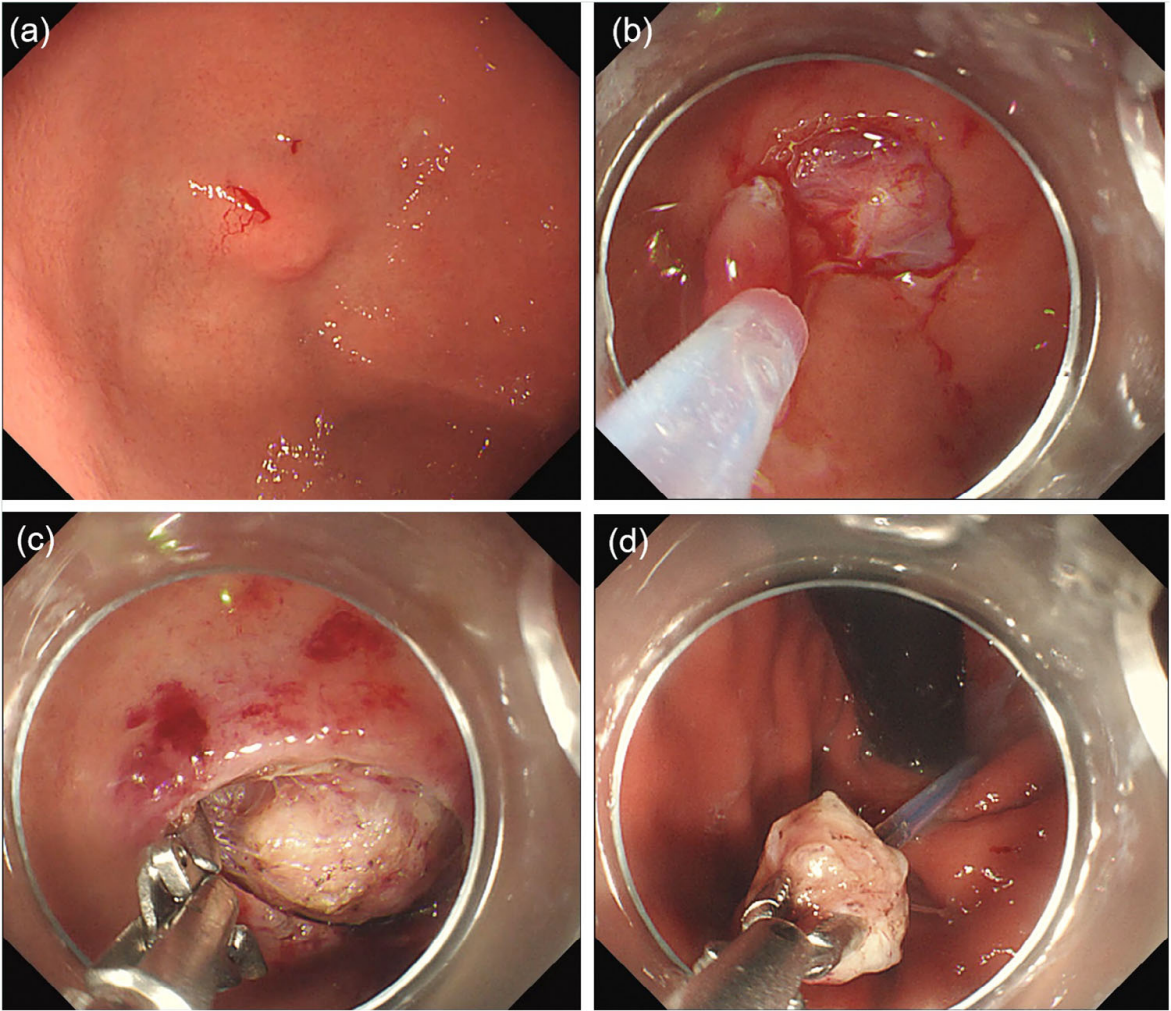
Endoscopic resection of gastric fundus submucosal tumors (a) A submucosal tumor in the fundus of the stomach. (b) The lesion surface mucosa was resected by a cold snare. (c) Hemostatic forceps were used to separate the submucosal connective tissue around the lesion. (d) The tumor was removed in a full layer by the snare.

A 56‐year‐old male patient was diagnosed with an SMT in the fundus of the stomach during a routine examination (Figure 1a). The SMT can be touched and slippable with a size of about 10 × 10 mm. Consequently, the patient sought endoscopic intervention at our department. Preoperative ultrasonography indicated that the lesion originated from the muscularis propria layer and exhibited hypoechoic characteristics, with a maximum cross‐section of about 10.7 × 6.2 mm (Video S1). Employing a novel resection technique that obviates the need for mucotomy knives and endoscopic injection needles, we initiated the procedure (Video S1). First, the lesion surface mucosa was resected by a cold snare to expose the tumor (Figure 1b).In the second step, hemostatic forceps were used to separate the submucosal connective tissue around the lesion by electrocoagulation and fully expose the tumor (Figure 1c).In the third step, the snare was placed over the tip of the endoscope and sent into the stomach with the endoscope, the tumor was seized by the forceps, the snare was released, and the tumor was removed in a full layer (Figure 1d). In the fourth step, use titanium clips to close the wound (Video S1). Upon meticulous examination of the postoperative specimens, it was determined that the total resection specimens exhibited a high degree of completeness (Video S1). The pathology report of the resected specimen suggested a low risk of GIST.

## DISCUSSION

Gastric SMTs are common and managing them has been challenging. There is growing interest in using endoscopy for both diagnosis and treatment. Recent studies have found that endoscopy ultrasonography with fine‐needle aspiration is the best method for assessing conditions like c‐KIT analysis and determining the mitotic index. Tumors larger than 2 cm should be carefully assessed for cancer, especially if fine‐needle aspiration is suggested.^6^ Leiomyomas smaller than 1cm can be followed up.^7^ Minor GISTs measuring less than 1cm in size are typically benign and can be safely resected via endoscopic procedures.^8^

Gastric fundus SMTs are difficult to resect with traditional ESD. Accessing lesions in the gastric fundus can be difficult during ESD due to its unique anatomical location, making the procedure more challenging.^9^ We report a case of expedited tumor resection achieved without the use of conventional ESD tools, such as a mucotomy knife and endoscopic injection needle, leading to significant cost reduction for the patients. Notably, the entire procedure was completed in a mere 19 min and was free of any untoward incidents.

Despite its numerous benefits, there are important considerations to keep in mind when implementing this new technology. Initially, we chose gastric fundus SMTs based on their size. Tumors larger than 1 cm were challenging to grasp with the 1‐cm mouse‐tooth forceps we used. Furthermore, Whether this method is useful not only for the intraluminal growth type but also for the extraluminal growth type will need to be investigated by accumulating more cases in the future. Ultimately, we believe that there is potential for enhancement in this technology, such as utilizing scissor‐type knives for trimming around the lesion in a manner that is both effective and minimally damaging to the tumor. By opting for larger mouse‐tooth forceps, the capability to resect larger tumors using this method is increased.

## CONFLICT OF INTEREST STATEMENT

None.

## PATIENT CONSENT STATEMENT

N/A.

## Supporting information

VIDEO S1 A novel technique for endoscopic resection of gastric fundus SMT.
